# Self-Reported Neck and Low Back Disability in Female Show Jumping Riders: A Cross-Sectional Study

**DOI:** 10.3390/jcm15155854

**Published:** 2026-07-27

**Authors:** Katarzyna Adamczewska, Mateusz Grajek, Tomasz Jurys, Ewa Malchrowicz-Mośko, Joanna Małecka, Marta Flis-Masłowska, Piotr Kocur

**Affiliations:** 1 Department of Neuromuscular Physiotherapy, Poznan University of Physical Education, 61-871 Poznań, Poland; malecka@awf.poznan.pl; 2 Department of Public Health, Faculty of Public Health in Bytom, Medical University of Silesia in Katowice, 40-055 Katowice, Poland; mgrajek@sum.edu.pl; 3 Department of Rehabilitation, Faculty of Health Sciences in Katowice, Medical University of Silesia in Katowice, 40-055 Katowice, Poland; tomasz.jurys@sum.edu.pl; 4 Department of Physical Education, University of Physical Education in Warsaw, 00-968 Warszawa, Poland; ewa.malchrowicz@awf.edu.pl; 5 Department of Kinesiotherapy Developmental Physiotherapy, Poznan University of Physical Education, 61-871 Poznań, Poland; flis-maslowska@awf.poznan.pl; 6 Department of Musculoskeletal Rehabilitation, Poznan University of Physical Education, 61-871 Poznań, Poland; pkocur@awf.poznan.pl

**Keywords:** show jumping, lumbar disability, cervical disability, Oswestry disability index, neck disability index

## Abstract

**Background**: Show jumping involves repetitive biomechanical loading of the rider’s spine. This study compared lumbar and cervical spine- related disability between amateur and advanced female show jumpers. **Methods**: This cross-sectional observational study included 353 female show jumping athletes aged 12–25 years. Data were collected using an anonymous online survey distributed during major international equestrian competitions in Poland. Participants were stratified by age (12–17 and 18–25 years) and competitive level (amateur vs. advanced riders) to examine differences in lumbar and cervical spine-related disability. Data were analyzed using non-parametric statistical tests, including the Mann–Whitney U test for continuous variables and the Chi-square test for categorical comparisons, with statistical significance set at *p* < 0.05. **Results:** Advanced athletes reported higher ODI scores compared with amateurs, particularly in the younger age group, although this did not reach statistical significance (*p* = 0.053). No significant differences were observed in the older age group. Similarly, no significant differences were found between amateur and advanced athletes regarding cervical spine-related disability (NDI) in either age group. Advanced athletes in both age groups reported significantly greater weekly training volume and longer training experience. Body mass did not differ between groups. **Conclusions**: Competitive level is associated with higher training loads, which may contribute to lumbar spine-related disability in younger female show jumpers. Further longitudinal studies are needed to explain the impact of cumulative loading on spinal health and to develop targeted preventive strategies.

## 1. Introduction

Chronic musculoskeletal issues, particularly low back pain (LBP), represent the most frequent overuse injuries among equestrian athletes [1]. Epidemiological data demonstrate that the prevalence of LBP in this sports discipline is high, often surpassing the rates observed in other athletic populations and the general public [2,3]. Given this pervasive nature of LBP, there is a clear imperative to investigate this phenomenon specifically within the younger age group.

Competitive riders are susceptible to severe training overloads, spinal microtraumas and functional disabilities [4]. One of the causes of horse riders’ back disability episodes may be the forced position in the saddle while riding. While shortened stirrups help athletes absorb forces and synchronize with the horse during jumps, this sustained multi-joint flexion may impair muscle length and flexibility [5].

During horseback riding, the show jumper’s upper body typically bends forward, and the head is positioned upward, looking for the direction of movement. It can cause muscle stiffness and disturb postural balance [6]. Higher training volumes and longer training experience characteristic of advanced athletes may produce cumulative mechanical loading of the spine region, potentially increasing the risk of functional disability [7]. The major muscle groups used by showjumpers during riding include mainly the hip adductors, erector spinae and rectus abdominis. The erector spinae and rectus abdominis are responsible for maintaining posture into the saddle, while hip adductors allow contact and guide the horse [8]. Weeks et al. (2024) confirmed that core stabilization and hip straightening exercises may be an effective method to reduce low back pain (LBP) in equestrians [9].

Horse riding transfers repetitive, compressive and torsional loads onto the rider [10]. Younger advanced equestrians appear to be at greater risk of developing spinal disabilities due to having more intense training sessions or less advanced riding technique than older riders [11]. Almost half of the child equestrian athletes (12–17 years old) suffered an episode of low back pain within the last 12 months [12]. This high prevalence underscores the need for monitoring and determining how chronic equestrian strains impact the athletes’ spine disabilities.

Neck Disability Index and Oswestry Disability Index are commonly used to measure functional disorders of the cervical and lumbar spine [13,14,15]. Despite the widespread use of these instruments, comparative data examining spinal disability across different age groups and competitive levels in female show jumping athletes remain scarce.

Regarding the application of these questionnaires in pediatric and adolescent populations (under 18 years of age), the literature presents a dual perspective. On one hand, several studies have successfully demonstrated that both the NDI and ODI exhibit high internal consistency, strong test–retest reliability, and clinical responsiveness in younger cohorts. Specifically, the NDI has been validated as a reliable tool for adolescents, as its core items—such as reading, concentration, and headaches—align well with the daily and academic activities of younger populations [16,17]. Similarly, the ODI has shown good psychometric value and sensitivity to clinical changes when evaluating adolescent populations with spinal pain [18,19]. On the other hand, a methodological debate persists regarding certain adult-specific domains, particularly within the ODI (e.g., items addressing driving or sex life), which may be less relevant or differently interpreted by minor respondents [20,21].

Nevertheless, in the absence of universally accepted, age-specific alternatives for athletic populations, both the NDI and ODI remain widely accepted and valuable screening tools in sports medicine, provided that age-dependent variations are carefully considered during data interpretation.

Most of the published studies focused on lumbar disabilities. However, the showjumping position during horseback riding may also lead to substantial overload in the cervical spine. Therefore, the objective of this cross-sectional observational study was to compare lumbar and cervical spine-related disability between amateur and advanced female show-jumping athletes across two age groups, while describing their respective training characteristics.

The primary outcome of this study was to compare the severity of self-reported cervical and lumbar spinal disability between amateur and advanced competitive female show jumpers, using NDI and ODI outcomes. We hypothesized *a priori* that advanced athletes, characterized by higher training volume and greater riding experience, would report higher levels of spinal disability compared to amateur athletes. Secondary descriptive variables were included to characterize the cohort and provide context for the primary between-group comparisons.

## 2. Materials and Methods

### 2.1. Study Design

This cross-sectional observational study evaluated lumbar and cervical spine-related disability in female show-jumping athletes. Data collection was centered around major indoor equestrian competitions in Poland, specifically the Cavaliada Tour and the FEI (*Fédération Équestre Internationale*) Jumping World Cup Central European League (CSI4*—Grand Prix, obstacle height 155 cm). The survey was distributed via a publicly accessible electronic link hosted on the official Cavaliada website. The questionnaire remained active for a total of one month, opening two weeks prior to the event and closing two weeks after its completion. To ensure population validity and isolate active competitive athletes, the survey included explicit self-report screening questions regarding competitive status, age, and discipline. During the post-collection data-cleaning phase, all responses from individuals who did not meet the strict inclusion criteria were filtered out and excluded from the final analysis.

### 2.2. Subjects

Age stratification allowed for a more accurate evaluation of the association between competitive level, training exposure, and spinal disability across developmentally distinct populations, while accounting for the methodological limitations of the ODI in adolescent cohorts [14]. Furthermore, this approach accounts for the unique risks associated with youth athletic development, cumulative training load, and overuse injuries, which are critical factors in the emergence of spinal disability in young athletes [22,23,24,25]. Participants were classified as advanced or amateur show jumping athletes based on their self-reported competitive level and regularity of participation in official competitions. Advanced riders were defined as athletes who regularly competed in national and international show jumping events, including FEI-sanctioned competitions, and who participated in classes with obstacle heights exceeding 130 cm. This level of competition reflects a higher technical demand, greater training intensity, and increased mechanical loading during riding and jumping activities. Amateur riders were defined as athletes competing primarily in lower-level regional or national events, with obstacle heights up to 120 cm, and without regular participation in high-level national or international competitions. This classification was chosen to distinguish riders with substantially different training volumes, technical demands, and cumulative exposure to sport-specific mechanical loads.

### 2.3. Data Collection

The initial sample size was calculated using a standard formula for a 95% confidence interval (CI) and a 5% margin of error, which yielded a minimum requirement of 384 respondents. To ensure sufficient statistical power and account for potential incomplete responses, a total of 400 questionnaires were systematically distributed to competitive female show jumpers and all 400 questionnaires were returned, yielding a response rate of 100%. Following a data screening process, 47 surveys were excluded due to missing values or incorrect completion. Consequently, 353 questionnaires were fully completed and qualified for final statistical analysis, resulting in a completion rate of 88.25%. The final sample included 205 amateur and 148 advanced athletes across both age cohorts. We did not observe statistically significant differences between the groups; however, given the exploratory nature of this study, these findings should be interpreted with caution, as the sample size may have been insufficient to detect smaller effect sizes. Given the scarcity of baseline data for this specific athlete population, a formal *a priori* power analysis based on effect sizes was not feasible at the study design stage. Therefore, the findings should be interpreted with awareness of the potential limitations regarding statistical power, and future studies with larger cohorts are encouraged to build upon these results.

### 2.4. Inclusion and Exclusion Criteria

Eligibility criteria included female sex, active participation in show jumping competitions, and age 12–25 years. No exclusion criteria were applied regarding current pain, prior injuries, or weekly training volume to ensure a representative sample of competitive athletes. Participation was anonymous and voluntary.

### 2.5. Assessment Tools

An anonymous online survey was used to assess spinal disability in female show jumpers. This method was selected to reduce time demands, minimize costs, and limit potential transcription errors associated with paper-based questionnaires, in accordance with recommendations for online research methodologies [26].

The survey was divided into two sections and required approximately five minutes to complete.

- Demographics and sport-specific variables: age, height, body mass, weekly training volume, and training experience (years of participation in show jumping).
- Outcomes: Spinal disability was evaluated using the validated Polish versions of the Oswestry Disability Index (ODI) for the lumbar spine and the Neck Disability Index (NDI) for the cervical spine [27,28]. ODI evaluates limitations across domains including pain intensity, personal care, lifting, walking, sitting, standing, sleeping, social life, and traveling, with total scores ranging from 0 to 50 [29].

Both questionnaires consist of 10 items, with each item scored from 0 to 5, yielding a maximum raw score of 50 points. For the ODI, raw scores were converted into percentages (0–100%) according to the official scoring guidelines. Disability categories for the ODI were defined as follows: minimal disability (0–20%), moderate disability (21–40%), and severe disability (41–100%). For the NDI, results were maintained as raw scores (0–50 points), with categories defined as: no disability (0–4 points), mild disability (5–14 points), moderate disability (15–24 points), and severe disability (25–34 points). In cases of missing data, questionnaires with more than one unanswered item were excluded from the analysis; for single missing items, the score was calculated based on the completed sections as recommended by the instrument guidelines. This methodological distinction in data presentation strictly follows the original validation guidelines established by the developers of each instrument. The same instruments and procedures were applied to all participants to ensure comparability between groups.

The internal consistency of the Polish versions of the ODI and NDI was evaluated using Cronbach’s alpha coefficient for each age stratum (12–17 and 18–25 years). This post-hoc analysis was performed to assess the reliability of these instruments within our specific cohort of equestrian athletes. Given that the ODI and NDI are primarily validated for clinical populations with significant spinal dysfunction, the reliability indices in this cohort of active athletes were interpreted with caution, taking into account the low variance of responses (ceiling effect) common in asymptomatic or low-disability populations.

### 2.6. Bias and Control

Efforts to reduce bias included using standardized, validated questionnaires (ODI and NDI) and applying the same online format to all participants. Potential confounding variables, including age, body mass, and training characteristics, were assessed through between-group comparisons. As no multivariable adjusted analyses were performed, the findings represent unadjusted between-group comparisons, and independent associations cannot be definitively determined.

### 2.7. Ethical Considerations

The study was conducted in accordance with the principles of the Declaration of Helsinki. The Bioethics Committee of Poznan University of Medical Sciences determined that formal ethical approval was not required for this study due to its non-experimental nature (Confirmation No. KB-208/26). Respondent consent was waived due to the implementation of a digital informed consent process, where adult participants provided consent via the ‘I agree’ option, whereas for volunteers aged 12–17, consent was obtained from their legal guardians via the ‘I agree with guardian’ option.

### 2.8. Statistical Analysis

All statistical analyses were performed using Statistica software (version 13; TIBCO Software Inc., Palo Alto, CA, USA) and Microsoft Excel (Microsoft Corp., Redmond, WA, USA). The normality of continuous variables was assessed using the Shapiro–Wilk test. The characteristics of the subjects were presented using counts and percentage frequencies for qualitative variables. Statistical tests (Mann–Whitney U test) were selected specifically to compare continuous data and Chi-square or Fisher’s exact test was used for categorical variables to provide a comprehensive profile of the groups, with the analysis prioritizing the primary hypothesis regarding competitive level. Subgroup analyses were conducted separately for each age group. Sensitivity analyses were performed by including incomplete surveys with imputed values for missing ODI or NDI items using mean substitution; results were consistent with the primary analyses. Statistical significance was set at *p* < 0.05. In addition to statistical significance, effect sizes were calculated using Cohen’s d to evaluate the magnitude of the observed differences. Furthermore, the clinical relevance of between-group differences was assessed in relation to previously reported minimal clinically important difference (MCID) values for the NDI and ODI scales.

## 3. Results

### 3.1. The Participant Selection Process Is Presented in Figure 1

The analysis included 174 riders younger than 18 years (70 advanced and 104 amateurs) and 179 riders aged 18–25 years (78 advanced and 101 amateurs).

**Figure 1:**
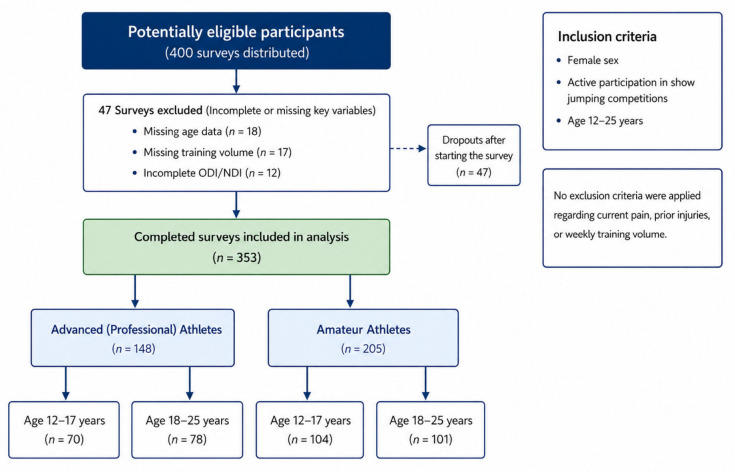
Flowchart of the study selection process.

### 3.2. Study Population and Clinical Characteristics Summarized in Table 1

Weekly time devoted to horse riding differed significantly between competitive levels in both age groups (*p* < 0.001). In riders younger than 18 years, amateur athletes most frequently reported training durations below 4 h per week (72%), whereas advanced athletes predominantly reported 4–10 h per week (69%). Training volumes exceeding 10 h per week were reported by 16% of advanced and only 2% of amateurs. A similar pattern was observed in the 18–25-year age group, where 65% of amateurs trained less than 4 h per week, while 65% of professionals reported 4–10 h of weekly riding. Training durations above 10 h per week were reported by 23% of advanced riders compared with 2% of amateurs.

Riding experience did not differ significantly between professional and amateur athletes in the younger age group (*p* = 0.076). In contrast, among athletes aged 18–25 years, riding experience differed significantly between competitive levels (*p* = 0.032), with a higher proportion of advanced athletes reporting more than 12 years of riding experience.

The distribution of NDI disability categories was comparable between advanced and amateur athletes in both age groups; in each group, mild disability was the most frequently reported category, whereas severe disability occurred rarely.

For ODI, the distribution of disability categories differed between competitive levels in athletes younger than 18 years, with advanced riders showing a higher prevalence of moderate and severe disability compared with amateurs. In the 18–25 age group, the prevalence of minimal disability in ODI was identical for both advanced and amateur riders (69%), with no statistically significant differences in the distribution of disability categories (*p* = 0.516).

In both age groups, body mass did not differ significantly between advanced and amateur athletes. Among riders younger than 18 years, mean body mass was 55.67 ± 9.85 kg in advanced and 57.53 ± 11.73 kg in amateurs (*p* = 0.532). Similarly, in the 18–25-year age group, body mass values were comparable between advanced riders (61.19 ± 10.86 kg) and amateurs (60.61 ± 9.64 kg; *p* = 0.897).

**Table 1:** Demographics, NDI and ODI outcomes and anthropometric characteristics among show jumping participants.

		12–17 Years				18–25 Years			
Variable	Category	Advanced (*n* = 70)	Amateur (*n* = 104)	Test Result	*p*-Value	Advanced (*n* = 78)	Amateur (*n* = 101)	Test Result	*p*-Value
Demographic and training characteristics									
*Riding experience* (years)	≤3	4 (5.7)	17 (16.3)	8.48 ^b^	0.076	2 (2.6)	6 (5.9)	10.56 ^b^	0.032
	4–7	37 (52.9)	57 (54.8)			13 (16.7)	29 (28.7)		
	8–11	23 (32.9)	27 (26.0)			28 (35.9)	42 (41.6)		
	12–15	5 (7.1)	3 (2.9)			27 (34.6)	17 (16.8)		
	>15	1 (1.4)	0 (0.0)			8 (10.3)	7 (6.9)		
*Weekly riding time* (h/week)	≤3	11 (15.7)	75 (72.1)	59.58 ^b^	<0.001	9 (11.5)	66 (65.3)	64.58 ^b^	<0.001
	4–10	48 (68.6)	27 (26.0)			51 (65.4)	33 (32.7)		
	>10	11 (15.7)	2 (1.9)			18 (23.1)	2 (2.0)		
Disability outcomes									
*Neck Disability* *Index (NDI)*	No disability	19 (27.1)	36 (34.6)	2.86 ^b^	0.414	26 (33.3)	27 (26.7)	2.86 ^b^	0.413
	Mild disability	38 (54.3)	53 (51.0)			42 (53.8)	63 (62.4)		
	Moderate disability	12 (17.1)	15 (14.4)			7 (9.0)	10 (9.9)		
	Severe disability	1 (1.4)	0 (0.0)			3 (3.8)	1 (1.0)		
*Oswestry Disability Index (ODI)*	Minimal disability	46 (65.7)	85 (81.7)	5.86 ^b^	0.053	54 (69.2)	70 (69.3)	1.32 ^b^	0.516
	Moderate disability	20 (28.6)	18 (17.3)			20 (25.6)	18 (17.8)		
	Severe disability	4 (5.7)	2 (1.9)			6 (7.7)	4 (4.0)		
Anthropometric characteristics									
*Body mass* (kg)	Mean ± SD	55.67 ± 9.85	57.53 ± 11.73	−0.62 ^a^	0.532	61.19 ± 10.86	60.61 ± 9.64	0.13 ^a^	0.897

Notes: ^a^ Mann–Whitney U test; ^b^ Likelihood ratio chi-square test. The values are presented as *n* (%), unless otherwise indicated. Continuous variables are presented as mean ± standard deviation (SD). Statistically significant *p*-values (*p* < 0.05) are indicated in bold. Abbreviations: NDI, Neck Disability Index; ODI, Oswestry Disability Index.

### 3.3. Spine Disability Outcomes (Distribution of Disability Categories Presented in Figure 2)

In athletes younger than 18 years, no significant differences were observed between advanced and amateur riders in NDI scores (9.08 ± 5.30 vs. 8.23 ± 5.66 points, respectively; *p* = 0.223; Cohen’s d = 0.15, 95% CI: −0.15 to 0.46). The effect size was very small. Advanced athletes had higher ODI values than amateur athletes (16.46 ± 12.61% vs. 12.33 ± 10.39%, respectively); however, the difference did not reach statistical significance (*p* = 0.0534). The corresponding effect size was small (Cohen’s d = 0.36, 95% CI: 0.06 to 0.67).

Among athletes aged 18–25 years, neither NDI nor ODI scores differed significantly between advanced and amateur athletes. Mean NDI values were 7.92 ± 6.21 points in advanced riders and 7.89 ± 4.98 points in amateurs (*p* = 0.509; Cohen’s d = 0.01, 95% CI: −0.29 to 0.30), indicating virtually no difference between the groups. Mean ODI values were 17.56 ± 14.64% and 15.13 ± 11.74%, respectively (*p* = 0.464; Cohen’s d = 0.19, 95% CI: −0.11 to 0.48), corresponding to a very small effect.

**Figure 2:**
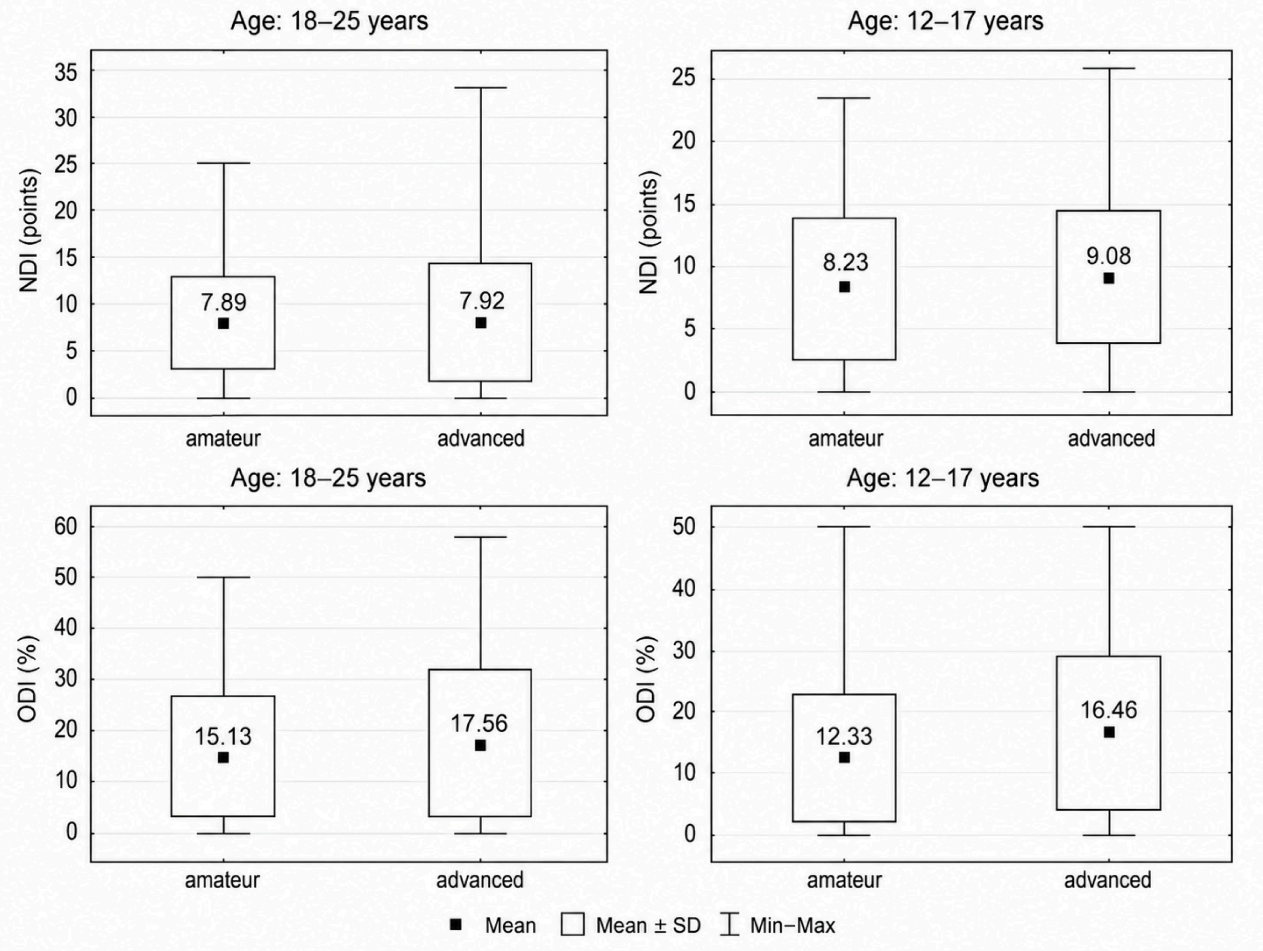
Mean (±SD) and range (min-max) of Neck Disability Index (NDI) and Oswestry Disability Index (ODI) scores in advanced and amateur equestrian athletes. Note: The boxes represent the mean ± standard deviation and represent the minimum and maximum values, rather than quartile-based interquartile ranges.

## 4. Discussion

Although observed effect sizes for NDI and ODI were small (Cohen’s d ranging from 0.01 to 0.36) and fell below the established MCID thresholds, these findings suggest that differences in spinal disability levels between advanced and amateur competitive riders are not clinically meaningful in the studied age groups. It should be noted that our study was underpowered to detect small effect sizes (below d = 0.42–0.44). Therefore, the absence of statistically significant differences between the groups does not imply a true absence of clinical differences; rather, our findings are compatible with potential effects up to the upper bounds of the reported confidence intervals. Presented findings regarding functional spinal disability may indicate that the mechanical loading of the lumbar region is already prominent during the early stages of an athlete’s career, demanding early screening and preventative strategies in adolescent riders. Adolescence is characterized by ongoing musculoskeletal maturation and evolving neuromuscular control, which may increase susceptibility to cumulative mechanical loading during intensive training [30]. Rapid somatic growth induces a transient disruption in motor coordination and proprioception [31]. Furthermore, recent evidence highlights that tissue adaptability and physical performance responses fluctuate significantly across different biological maturation stages. When intensive training loads overlap with these vulnerable developmental windows, the mismatch between structural bone growth and muscle-tendon force-generating capacity drastically limits the tolerance to repetitive mechanical strain, thereby elevating the risk of cumulative microtrauma [32].

Low back disability represents one of the most prevalent musculoskeletal complaints among equestrian athletes and constitutes a major factor limiting performance and long-term participation in the sport [33]. The specific biomechanical demands of horseback riding, particularly in show jumping, expose riders to repetitive axial loading, impact forces, and complex trunk–pelvis interactions that may contribute to the development of self-reported lumbar disability. Competitive participation is typically associated with higher training volumes and longer riding experience, which may be associated with cumulative mechanical loading of the lumbar spine [34].

In contrast, the influence of competitive riding on cervical spine-related disability remains less clearly defined [35]. Therefore, the present study aimed to investigate the association between competitive level, training exposure, and lumbar and cervical spine-related disability in female show-jumping athletes across two age groups. The more pronounced differences in lumbar spine-related disability observed in younger athletes may be partly explained by age-related developmental factors. These results may suggest an association between intensive competitive exposure during this developmental period and reported lumbar spine symptoms in young female show jumpers.

Although no statistically significant differences in ODI scores were observed between competitive levels, the presence of reported lumbar disability among young, active athletes is a finding of potential clinical interest. In a population where optimal musculoskeletal health is expected, the occurrence of any functional disability warrants attention. Our findings suggest that spinal complaints are not confined to advanced athletes but may be present across the spectrum of young show jumping participants. This observation aligns with the broader literature; for instance, Kraft et al. [10] demonstrated that the prevalence of low back symptoms within the equestrian population is over 50% higher than in the general population. Horseback riding is a unique discipline characterized by a dynamic interaction between the rider and the animal. Consequently, numerous factors beyond the horse’s gait and carrying capacity contribute to LBP in riders. Therefore, there is a need to better understand the specific predictors that increase mechanical loads on the low back, including poor equestrian technique, incorrect riding posture, and anthropometric mismatches. Recent biomechanical studies suggest that peak acceleration and shock attenuation might play a role in chronic low back pain among equestrian athletes [36].

Functional movement adaptations specific to equestrian disciplines have also been reported. Lewis et al. (2009) identified discipline-specific differences in postural control and trunk stability among female riders, suggesting that these adaptations may influence loading patterns in both the lumbar and cervical spine [37].

The clinical relevance of the observed between-group differences was assessed in relation to previously reported minimal clinically important difference (MCID) values. Published MCID estimates vary depending on the population and methodology, but values of approximately 5–8 points on the 0–50 NDI scale and approximately 10 percentage points for the ODI are commonly reported. In the present study, the largest between-group differences were 0.85 points for the NDI and 4.13 percentage points for the ODI. Therefore, none of the observed mean differences exceeded commonly reported MCID thresholds, suggesting that the differences between advanced and amateur riders were likely limited in clinical magnitude. However, MCID values are primarily intended to interpret longitudinal within-person changes, particularly following treatment, rather than cross-sectional differences between independent groups [38,39]. Moreover, no MCID values have been established specifically for young female show-jumping athletes. Therefore, these thresholds were used only as approximate contextual benchmarks. To further interpret our findings, we conducted a sensitivity analysis to determine the minimum effect size detectable with our sample size (d = 0.42–0.44 at 80% power). This analysis, when considered alongside the Minimal Clinically Important Difference (MCID) for the ODI and NDI, provides a comprehensive framework for evaluating our results. While the observed effect sizes fell below our sensitivity threshold, they also did not consistently reach the threshold of the MCID reported in previous clinical literature. Together, these observations suggest that the absence of statistical significance in our cohort should be interpreted not as definitive evidence of equality, but as a result of the study being underpowered to detect smaller, potentially clinically relevant variations.

In conclusion, our findings highlight that self-reported spine disability may be a noteworthy issue among young show jumping athletes, which appears to be independent of competitive level. The observed association between higher training volumes and lumbar symptoms, particularly during developmental stages, underscores the need for a shift in focus toward long-term spinal health monitoring. Future research should prioritize longitudinal designs to better disentangle the effects of cumulative mechanical loading from developmental factors. Furthermore, to overcome the limitations of the present study regarding statistical power, future investigations should aim for larger sample sizes. This will enhance the ability to detect subtle clinical variations and ultimately aid in the development of targeted, evidence-based conditioning and preventive programs for adolescent riders.

### 4.1. Limitations

Several limitations of our study merit consideration. The primary limitation arises from its nature. While we used standardized, validated tools such as ODI and NDI to reduce assessment bias, the use of an online survey introduces selection bias. Another significant limitation pertains to our reliance on unadjusted comparative analyses, which precludes the definitive establishment of independent associations between training-related variables and spinal disability outcomes. The absence of diagnostic imaging limits our ability to correlate subjective disability scores with underlying structural spinal pathology, such as disc degeneration or structural damage, which are known to be significant contributors to chronic back pain [40]. Finally, the external validity of our findings is limited by the study’s specific sampling framework. As data collection was centered on indoor, high-level show jumping events in Poland, our sample may not be representative of show jumpers competing in different environmental conditions or in other international regions with varying training cultures. Future multi-center, cross-cultural studies are necessary to determine the generalizability of these findings to a broader, global population of equestrian athletes.

### 4.2. Recommendations

Regular monitoring of spinal health using standardized instruments (ODI/NDI) is recommended for competitive show jumpers, as our findings indicate that spinal discomfort occurs across both age groups and training levels. Our findings support the implementation of early preventive care and regular physical assessments for equestrian athletes. For example, combined exercise protocols, such as those integrating yoga and back-school programs, have demonstrated effectiveness in managing chronic non-specific low back pain in clinical trials and represent a practical candidate approach for future preventive protocols in this population [41].

## 5. Conclusions

In summary, our findings indicate that spinal disability is a relevant issue among young, competitive show jumping athletes, regardless of their training level. Given that the developmental age is a critical period for spinal health, the implementation of proactive monitoring protocols is recommended. Future studies should focus on longitudinal observations and multivariable analyses to better understand the mechanisms of spinal impairment and to develop evidence-based preventive strategies tailored to this specific population.

## Data Availability

The data supporting the findings of this study are available from the corresponding author upon reasonable request.
